# IgA Vasculitis Associated With COVID-19

**DOI:** 10.7759/cureus.38725

**Published:** 2023-05-08

**Authors:** Yousef Salem, Zaryab Alam, Mojahed M Shalabi, Gregory A Hosler, Sampada Acharya

**Affiliations:** 1 Department of Dermatology, University of Texas Health Science Center at San Antonio, San Antonio, USA; 2 Department of Dermatology, Texas A&M College of Medicine, Bryan, USA; 3 Department of Internal Medicine, Baylor Scott & White All Saints Medical Center, Fort Worth, USA; 4 Department of Dermatology, University of Texas Southwestern Medical School, Dallas, USA; 5 Department of Rheumatology, Baylor Scott & White All Saints Medical Center, Fort Worth, USA

**Keywords:** vasculitis, dermatopathology, covid-19, henoch-schonlein purpura, iga vasculitis

## Abstract

IgA vasculitis, also known as Henoch-Schonlein Purpura (HSP), is an inflammatory disorder of small blood vessels that can present with palpable purpura, arthralgias, abdominal pain, and kidney disease. It is most commonly found in pediatric patients after an inciting infection but has been seen across all ages and associated with certain drugs and vaccines. COVID-19 has been associated with various cutaneous manifestations, but HSP is a rarely reported one.

We present a case of a 21-year-old female presenting with a petechial rash found to be seronegative IgA vasculitis presenting concurrently with dyspnea secondary to COVID-19. She was initially seen by an outside practitioner, tested negative for COVID, and was prescribed a course of oral prednisone. Shortly thereafter, she visited the ED for worsening shortness of breath and tested positive for COVID-19, for which she received Paxlovid. Biopsy after a visit to a dermatologist confirmed intramural IgA deposition on immunofluorescence, and she was tapered off prednisone and started on azathioprine.

## Introduction

Several dermatoses have been associated with COVID-19, with the most common presentations including pseudo-chilblains, maculopapular, urticarial, vesicular, and vaso-occlusive [1]. IgA vasculitis, also known as Henoch-Schonlein Purpura (HSP), is a small vessel vasculitis that often presents following viral infections, most commonly appearing as a petechial rash. We describe an unusual case of HSP associated with COVID-19 infection.

## Case presentation

**Figure 1:**
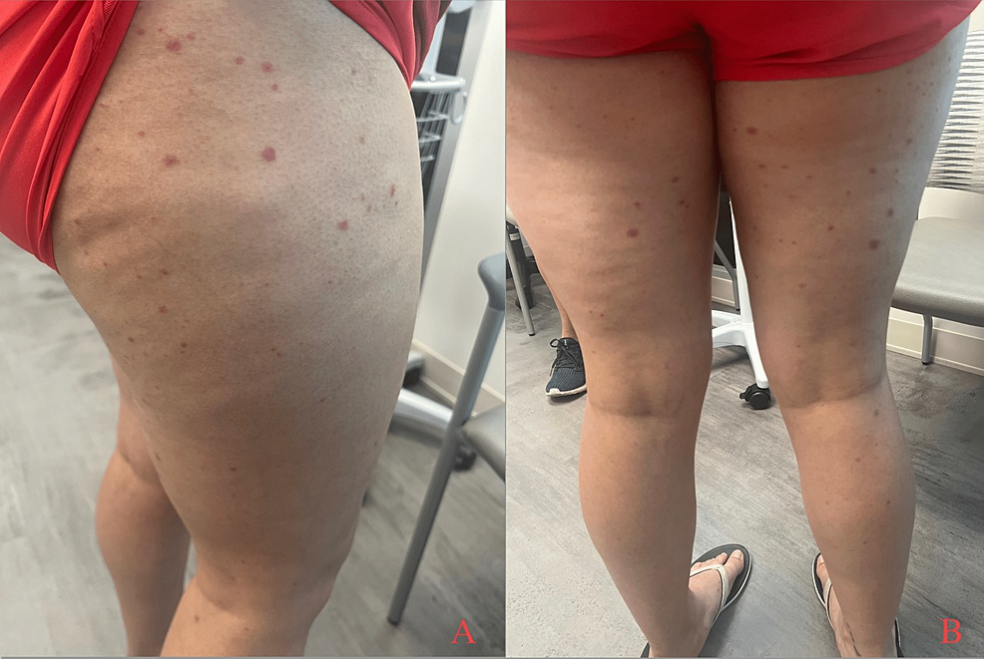
Lateral view (A) and posterior view (B) of multiple scattered raised petechial lesions seen on gross exam of the patient's upper legs

A 21-year-old female with no history of inflammatory arthritis or skin psoriasis presented to the clinic with a morbilliform, petechial rash without pain or pruritus on her legs, feet, back, and forearms slightly improved with ibuprofen (Figures 1A, 1B). She stated that it initially appeared on her inner thighs before spreading while she was vacationing in Florida. She also had experienced shortness of breath that limited her ability to walk as well as night sweats, fatigue, and diarrhea, but initially tested negative for COVID-19. Seven days later, she was seen by her primary care physician with worsening dyspnea and was sent to the emergency department where she was found to be COVID-19 positive, for which she received a seven-day course of Paxlovid. She denied any fever, hematuria, hemoptysis, or anosmia. She was prescribed prednisone to be taken after a scheduled biopsy.

**Figure 2:**
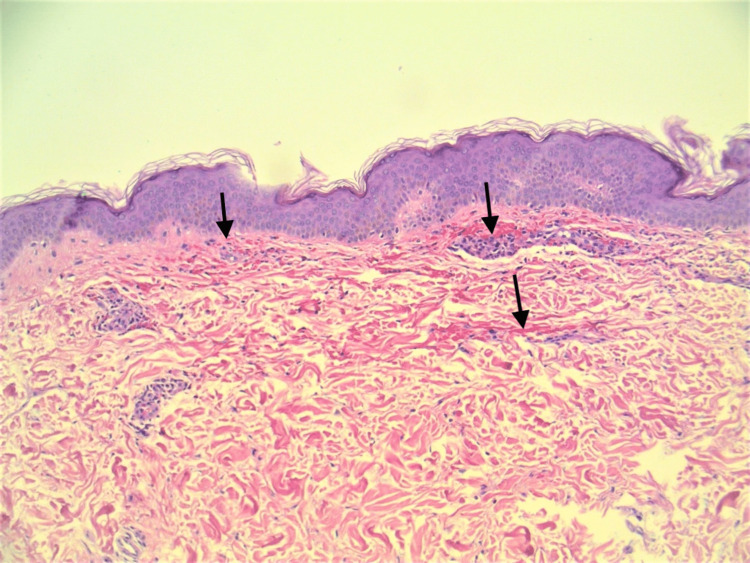
Medium power image showing perivascular extravasation of red blood cells and neutrophils into interstitial space

**Figure 3:**
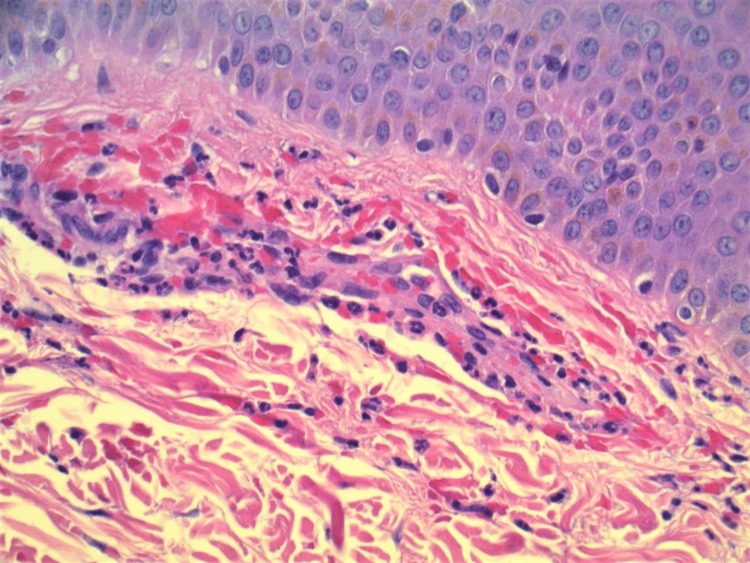
High power image showing perivascular neutrophilic infiltrate in superficial dermis with extravasated erythrocytes

Biopsy taken at a later visit showed mild perivascular neutrophilic infiltrate in the superficial dermis with extravasated erythrocytes (Figures 2, 3). Immunofluorescence was positive for intramural deposition of IgA within small caliber vessels, and negative for fibrinogen, IgG, C3, and 19M. Serum C3 was mildly elevated, and C-reactive protein (CRP) and erythrocyte sedimentation rate were increased, suggesting an inflammatory disease. Markers for vasculopathies including serum antinuclear antibody (ANA), rheumatoid factor (RF), SS-A, SS-B, Epstein-Barr virus (EBV), and antineutrophilic cytoplasmic antibody (ANCA) were negative. A chest x-ray did not indicate lung involvement, and neither did a computed tomography (CT) scan of the chest. However, CT did show evidence of fatty liver, and a complete metabolic panel demonstrated increased liver function tests (LFT) aspartate aminotransferase (AST) and alanine aminotransferase (ALT). Urinalysis showed only trace protein, and creatinine was unremarkable. The patient was prescribed azathioprine as a steroid-sparing agent, and her previously prescribed steroids were tapered off.

## Discussion

This was an interesting case of IgA vasculitis temporally correlated with COVID-19 in the absence of other associated infections. As more patients are diagnosed with COVID-19, we anticipate an increase in cases involving HSP associated with COVID-19. While HSP is often self-limited, a small proportion of patients experience long-term effects such as joint pain and stiffness, renal damage, and gastrointestinal bleeding. In severe cases, it can lead to chronic renal disease and even renal failure [2].

To evaluate a patient with IgA vasculitis and COVID-19, renal tests such as urinalysis, serum creatinine, glomerular filtration rate (GFR), and biopsy may be used to assess kidney function and damage [3]. Monitoring for changes in kidney function is crucial as it can be a serious complication.

The mainstay of treatment in uncomplicated cases is non-steroidal anti-inflammatory drugs (NSAIDs) to reduce inflammation and pain [4]. Corticosteroids have been shown to shorten the length of abdominal pain symptoms but do not otherwise affect the disease course, at least in pediatric patients. If renal involvement is suspected, the use of angiotensin-converting enzyme inhibitors (ACEi) is recommended [5]. In more severe cases, high-dose corticosteroids, immunosuppressants, plasma exchange, or intravenous immunoglobulin may be used [6].

One proposed mechanism of COVID-19-induced HSP is direct damage to blood vessels by the virus followed by the formation of immune complexes and an inflammatory response. Another proposed mechanism is the viral induction of a systemic inflammatory response that triggers HSP development. Cases of IgA vasculitis following vaccination for COVID-19 have also been described, indicating a possible involvement of the spike protein used in some vaccines [7]. The exact mechanism linking COVID-19 to HSP is still under investigation, and further research is necessary to elucidate this connection.

HSP following COVID-19 is an uncommon occurrence, with most cases of HSP appearing in children (between three and 26.7 cases per 100,000 population) [8]. A systematic review conducted in 2022 showed 36 cases of vasculitis following COVID-19 in pediatric patients, of which nine were IgA and six of which did not have any pre-existing co-morbidities [9]. Another case was described by Allez et al. in a 24-year-old patient; however, he had a known history of Crohn's disease and was taking immunosuppressant medication [10]. Further research may analyze risk factors, differences in presentation, and effective management for COVID-19-associated IgA vasculitis as compared with cases caused by other agents.

## Conclusions

IgA vasculitis, also known as HSP, should be considered in patients who develop a pathognomonic rash after a COVID-19 infection. Early recognition and appropriate management of IgA vasculitis are crucial to prevent long-term complications and improve patient outcomes. A thorough work-up, including a complete metabolic panel and involvement of nephrology, is necessary to evaluate for renal involvement. Future studies with larger sample sizes are recommended to expand on the relationship between COVID-19 and vasculitis.

## References

[1] Tan SW, Tam YC, Oh CC (2021). Skin manifestations of COVID-19: a worldwide review. JAAD Int.

[2] Jithpratuck W, Elshenawy Y, Saleh H, Youngberg G, Chi DS, Krishnaswamy G (2011). The clinical implications of adult-onset Henoch-Schonelin purpura. Clin Mol Allergy.

[3] Guo D, Lam JM (2016). Henoch-Schönlein purpura. CMAJ.

[4] Tudorache E, Azema C, Hogan J (2015). Even mild cases of paediatric Henoch-Schönlein purpura nephritis show significant long-term proteinuria. Acta Paediatr.

[5] Al Harash A, Saeli S, Lucke M, Arora S (2020). IgA vasculitis nephritis: a case series and comparison of treatment guidelines. Case Rep Rheumatol.

[6] Abu-Zaid MH, Salah S, Lotfy HM (2021). Consensus evidence-based recommendations for treat-to-target management of immunoglobulin A vasculitis. Ther Adv Musculoskelet Dis.

[7] Grossman ME, Appel G, Little AJ, Ko CJ (2022). Post-COVID-19 vaccination IgA vasculitis in an adult. J Cutan Pathol.

[8] Oni L, Sampath S (2019). Childhood IgA vasculitis (Henoch Schonlein purpura)-advances and knowledge gaps. Front Pediatr.

[9] Batu ED, Sener S, Ozen S (2022). COVID-19 associated pediatric vasculitis: a systematic review and detailed analysis of the pathogenesis. Semin Arthritis Rheum.

[10] Allez M, Denis B, Bouaziz JD (2020). COVID-19-related IgA vasculitis. Arthritis Rheumatol.

